# Parenteral Antibiotics Reduce Bifidobacteria Colonization and Diversity in Neonates

**DOI:** 10.1155/2011/130574

**Published:** 2010-08-03

**Authors:** Séamus Hussey, Rebecca Wall, Emma Gruffman, Lisa O'Sullivan, C. Anthony Ryan, Brendan Murphy, Gerald Fitzgerald, Catherine Stanton, R. Paul Ross

**Affiliations:** ^1^Department of Paediatrics and Child Health, University College Cork, Cork, Ireland; ^2^Division of Gastroenterology, Hepatology and Nutrition, The Hospital for Sick Children, Toronto, ON, Canada M5G 1X8; ^3^Alimentary Pharmabiotic Centre, Cork, Ireland; ^4^Department of Biotechnology, Teagasc Moorepark Food Research Centre, Fermoy, Cork, Ireland; ^5^Department of Microbiology, University College Cork, Cork, Ireland

## Abstract

We investigated the impact of parenteral antibiotic treatment in the early neonatal period on the evolution of bifidobacteria in the newborn. Nine babies treated with intravenous ampicillin/gentamicin in the first week of life and nine controls (no antibiotic treatment) were studied. Denaturing gradient gel electrophoresis was used to investigate the composition of *Bifidobacterium* in stool samples taken at four and eight weeks. Bifidobacteria were detected in all control infants at both four and eight weeks, while only six of nine antibiotic-treated infants had detectable bifidobacteria at four weeks and eight of nine at eight weeks. Moreover, stool samples of controls showed greater diversity of *Bifidobacterium* spp. compared with antibiotic-treated infants. In conclusion, short-term parenteral antibiotic treatment of neonates causes a disturbance in the expected colonization pattern of bifidobacteria in the first months of life. Further studies are required to probiotic determine if supplementation is necessary in this patient group.

## 1. Introduction

Up to 10% of newborn infants require admission to the neonatal intensive care unit (NICU) for ongoing medical care [1–3]. Many of these infants require treatment with parenteral antibiotics for variable periods of time. Antibiotic administration is known to perturb the composition of the intestinal microbiota, resulting in suppression of anaerobic bacteria (with the exception of clostridia, which remain at detectable levels) and increased numbers of potentially pathogenic bacteria such as *Klebsiella*, *Enterobacter*, *Citrobacter,* and *Pseudomonas* [4, 5]. Since the pioneering microbiota of infancy probably create perpetual habitats for themselves, defining the life-long composition of the gut microbiota and consequently contributing to host health and well-being [6, 7], the initial colonization of the infant gastrointestinal tract is of significant importance. 

*Bifidobacterium* spp. have several reported health-promoting effects, including inhibition of growth of harmful bacteria, stimulation of the immune system, alleviation of constipation, and prevention of intestinal infections [8–13]. Species commonly associated with humans include *Bifidobacterium infantis, B. longum, B. bifidum, B. breve, B. catenulatum, B. pseudocatenulatum, B. angulatum, B. gallicum, *and *B. adolescensis* [8], of which the first four are prevalent in infants [14]. 

Suppression of the bifidobacteria population as occurred following antibiotic exposure in children [5, 15, 16] may have negative effects on host well-being and can be associated with higher susceptibility to enteropathogenic bacterial infection [17]. However, the impact, if any, of parenteral antibiotics prescribed shortly following birth on the developing bifidobacterial population has not been addressed. The aim of this study was therefore to determine the impact of intravenous antibiotic treatment in the neonatal period on the evolution of bifidobacterial colonization over time.

## 2. Material and Methods

### 2.1. Subjects

Faecal material was sampled, using swabs, from 18 infants, nine infants previously treated with antibiotics and nine healthy controls (Table 1). Approval for the study protocol was obtained from the Clinical Research Ethics Committee of the Cork Teaching Hospitals, Cork, Ireland. Inclusion criteria were defined as full or near term infants requiring parenteral antibiotic administration within the first 48 hours of life. Control subjects were eligible if they were full or near term infants who were otherwise well and did not require admission to the NICU. Infants placed on oral antibiotics, those kept *nil by mouth* or requiring surgery, infants with congenital anomalies, or those born before term were excluded from the study. The indications for antibiotic treatment were determined clinically, based on symptoms and/or signs of suspected sepsis, and independent of the study investigators. Written informed consent was obtained from parents of all infants. Stool samples were taken by the same clinician at four and eight weeks after birth and stored at −20°C pending analysis.

### 2.2. PCR -DGGE Analysis

Bacterial DNA was extracted from swabs using a QIAamp DNA stool minikit (Qiagen, Hilden, Germany) by following the manufacturer's instructions (lysis temperature, 95°C). To investigate the bifidobacterial population in the samples, PCR was performed as a nested approach. The first PCR applied primers Im26-f (5′- GATTCTGGCTCAGGATGAACG -3′) and Im3-r (5′- CGGGTGCTICCCCACTTTCATG -3′) described by Kaufmann et al. [18] amplified a 1,417 bp fragment of the bifidobacterial 16S rRNA gene. PCR volumes of 50 *μ*L contained 5 *μ*L of 10× PCR buffer, 5 *μ*L of MgCl_2_ (2.5 mM), 8 *μ*L of dNTPs (0,2 mM each), 1 *μ*L of each primer (5 pmol), 0.5 *μ*L of Taq polymerase (5 U/*μ*L), 28.5 *μ*L of sterile Milli-Q water, and 1 *μ*L of DNA solution. PCR-reagents were obtained from Bioline (Taunton, MA, USA). The following PCR program was used: initial denaturation at 94°C for 5 min; 3 cycles of denaturation at 94°C for 45 s, annealing at 57°C for 2 minutes, and extension at 72°C for 1 min; 30 cycles of denaturation at 94°C for 20 s, annealing at 57°C for 1 min, and extension at 72°C for 1 min; and final extension at 72°C for 5 min followed by cooling to 4°C. The presence of PCR products was verified on a 1.5% (w/v) agarose gel. In order to eliminate the remaining oligonucleotides and original template DNA, purification of the amplicons was performed by use of the QIAquick PCR purification kit (Qiagen) according to the manufacturer's instructions. A second PCR was performed using the amplicons of the first PCR as template DNA. The primer set used (F357-GC and 518R) (5′GC-clamp- GCCTACGGAGGCAGCAG -3′ and 5′- ATTACCGCGGCTGCTGG -3′, resp.) amplifies the V3 region of the bacterial 16S rRNA gene [19]. The forward primer contained a GC clamp (5′- CGCCCGCCGCGCGCGGCGGGCGGGGCGGGGGCACGGGGG -3′) to facilitate separation of the amplicons in a DGGE gel. PCR volumes of 50 *μ*L contained 5 *μ*L of 10× PCR buffer, 2 *μ*L of MgCl_2_ (1 mM), 8 *μ*L of dNTPs (0.2 mM each), 2 *μ*L of each primer (5 *μ*M), 0.5 *μ*L of Taq polymerase (5 U/*μ*L), 29.5 *μ*L of sterile Milli-Q water, and 1 *μ*L of 10-fold diluted DNA solution (obtained from the first PCR). The following PCR program was used: initial denaturation at 94°C for 5 min, 30 cycles of denaturation at 94°C for 20 s, annealing at 58°C for 45 s, and extension at 72°C for 1 min, and final extension at 72°C for 7 min, following by cooling to 4°C. PCR products were analyzed on DGGE gels based on the protocol by Temmerman et al. [10]. A Dcode universal mutation detection system (Bio-Rad Laboratories, Hercules, CA, USA) was used, utilizing 16 cm by 16 cm by 1 mm gels. Eight percent polyacrylamide gels were prepared and run with 1× TAE buffer diluted from 50× TAE buffer (2 M Tris base, 1 M glacial acetic acid, and 50 mM EDTA). The denaturing gradient was formed with two 8% acrylamide (acrylamide-bis/acrylamide 40%) stock solutions (Severn Biotech Ltd, Worcestershire, UK). A 100% denaturing solution contained 40% (v/v) formamide and 7.0 M urea. The gels were poured from the top by using a gradient maker (CBS Scientific Linear Gradient Maker, Bio-Rad Laboratories) and a pump (Bio-Rad Laboratories) and gradients of 50% to 70% were used for the separation of the generated amplicons. Before polymerization of the denaturing gel (24 mL gradient volume), a 6 mL stacking gel without denaturing chemicals was added, and the appropriate comb was subsequently inserted. PCR amplicons were separated by electrophoresis at a constant voltage of 60 V in a 0.5 × TAE buffer at a constant temperature of 60°C for 16 h. Gels were stained in ethidium bromide for 30 min, allowing digital capturing of the DGGE band profiles under UV light.

### 2.3. Reference Ladder

A mixture of five different species of bifidobacteria which are the most commonly isolated species of bifidobacteria from the infant gastrointestinal tract (GIT) [14, 20, 21] was used to create a reference ladder in order to enable a visual comparison of the bands. DNA extraction from the reference strains, *Bifidobacterium breve, B. bifidum, B. infantis, B. adolescentis, *and* B. longum,* was performed according to the method described by Hoffman and Winston [22].

## 3. Results

Nine subjects were recruited to both control and antibiotic-treated groups in this study. Their pertinent demographic data are summarized in Table 1. While the gender distribution and feeding history was similar in both groups, the antibiotic treated infants were more likely to be born by caesarean section (5/9 infants) and all control infants were born by spontaneous vaginal delivery. Ampicillin and gentamicin were the only antibiotics used and the median duration of therapy was 2 days (range 2–9 days). 

### 3.1. Development of the *Bifidobacterium* Population at Four Weeks of Age

In order to investigate the population of bifidobacteria in the samples, the *Bifidobacterium*-specific primers Im-3 and Im-26 were used. Amplicons obtained using these primers served as template DNA for the V3 primer combination V3R and V3F, during a second PCR step. The mobility of the PCR products obtained by these primers in DGGE was compared with the PCR pattern of bifidobacteria reference strains. Because of the high G+C content of bifidobacteria, the conventional 35% to 70% denaturing gradient was replaced with a 50% to 70% denaturing gradient.

**Figure 1:**
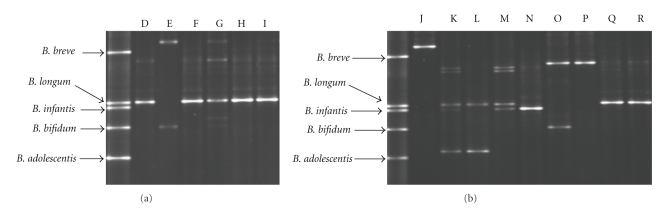
DGGE of bifidobacterial PCR-products (V3-region) from stool samples taken at four weeks of age from infants treated with antibiotics D–I (a) and controls J–R (b). The mobility of the PCR products obtained in DGGE was compared to the PCR pattern of reference strains obtained with the same primer set.

At four weeks of age, species of bifidobacteria were detected in six out of nine infants treated with antibiotics (subjects A–I) and in all infants in the control group (subjects J–R). The samples from the antibiotic treated infants exhibited a lower number of bands in the DGGE gel (Figure 1(a)) compared with controls (Figure 1(b)) at four weeks of age, representing a less diverse population of bifidobacteria at this age. Five of six infants in the antibiotic-treated group showed a single dominant band, which corresponded to *B. longum* (Figure 1(a)). All five infants had been treated with intravenous ampicillin/gentamicin for two days and there were both breast fed (subject F, G) and formula fed (subject D, H, I) infants within this group. The sample lacking a band in the position of *B. longum* was obtained from an infant that had received antibiotics for five days (subject E). According to the band pattern of this subject, some *Bifidobacterium* species were able to colonize this infant despite antibiotic treatment for five days; that is, the lower band appears to be *B. bifidum*, while the upper band is not comparable to any of the species within the reference ladder. All samples from the formula fed controls (K, L, M, Q, R) exhibited a band in the same position as *B. longum *at four weeks of age, except for one infant (subject J) (Figure 1(b)). In contrast, samples from the breast fed infants in the control group (N, O, P) showed bands in the positions of *B. infantis* and *B. bifidum. *These two species were only found in one of the formula fed infants. While all vaginally born infants in the antibiotic-treated group harboured bifidobacteria by four weeks of age, two out of five infants born by caesarean section harboured bifidobacteria (*B. bifidum* and *B. longum*) at this time point.

### 3.2. Development of the *Bifidobacterium* Population at Eight Weeks of Age

**Figure 2:**
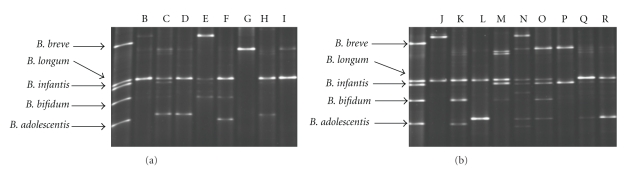
DGGE of bifidobacterial PCR-products (V3-region) from stool samples taken at eight weeks of age from infants treated with antibiotics B–I (a) and controls J–R (b). The mobility of the PCR products obtained in DGGE was compared to the PCR pattern of reference strains obtained with the same primer set.

At eight weeks of age, eight out of nine antibiotic-treated infants and all controls had detectable levels of bifidobacteria in their stools. Interestingly, the only sample in which bifidobacteria was not detected was from an infant that had received antibiotics for nine days (subject A) (the other infants were treated with antibiotics for 2–5 days). Between four and eight weeks, an increase in bifidobacteria diversity in both groups was observed as evidenced by the increased number of bands (Figure 2). Nonetheless, while both groups were similar at eight weeks, samples from antibiotic-treated infants continued to display a less diverse population of bifidobacteria when compared with controls (Figure 2). The samples obtained from the breast-fed infants in the control group had in general a more diverse population of bifidobacteria compared with formula-fed infants (7-8 bands compared with 2–5 bands, resp.). However, no differences between the samples from the breast-fed and formula-fed infants in the antibiotic-treated groups were observed. Subject G exhibited a decrease in diversity from four to eight weeks of age. Moreover, the band with highest intensity at four weeks, identified as *B. longum*, was replaced by another dominant strain, identified as *B. breve*, after eight weeks. Four of the five infants in the antibiotic-treated group born by caesarean section harboured bifidobacteria by week eight. As found at week four, all vaginally born infants harboured bifidobacteria at eight weeks of age, with a higher diversity observed compared with four weeks of age. Overall, *B. longum* appeared to be the most commonly detected *Bifidobacterium* species in the infants at eight weeks of life (present in 15 out of 17 infants harbouring bifidobacteria) (Figure 2).

## 4. Discussion

*Bifidobacterium *and *Lactobacillus* species are considered among the most important beneficial bacteria in the human GIT and their presence in large numbers in the microbiota is regarded as beneficial [23, 24]. Suppression of these beneficial components could facilitate growth of potentially pathogenic bacteria such as *Klebsiella *and *Clostridium* and have consequences for the well-being of the host. This study demonstrates that even brief parenteral antibiotic treatment alters the pattern of bifidobacterial evolution over time. Infants treated with antibiotics showed a reduction in both colonisation and diversity of bifidobacteria compared with controls. While control and antibiotic-treated groups were similar by eight weeks of age, a number of control subjects continued to display a more diverse population of bifidobacteria. Mackie et al. [25] reported that the colonisation of bifidobacteria in infants is normally well established by one week of age. It is tempting to speculate that the observed difference in detectable levels of bifidobacteria between the antibiotic-treated infants and controls in our study reflects the early antibiotic exposure. Favier et al. [26] previously described a significant difference in the microbiota of a single infant receiving antibiotics compared to four nonantibiotic treated infants, using similar methodology that is, PCR-DGGE and *Bifidobacterium*-specific primers as used in our study. *Bifidobacterium* spp. were absent in the antibiotic-treated infant at all time points assessed, from five days to five months of age. However, in that study, the antibiotic-treated subject received prolonged antibiotic therapy with both parenteral and oral agents (thirteen days of parenteral coamoxiclav followed by oral cotrimoxazole) [26]. In contrast, the majority of participants in our study had relatively brief periods of intravenous antibiotic administration and yet differences were still observed compared with controls.

*B. longum* was the dominant species in both the antibiotic-treated infants and in the control group. This finding is consistent with the study by Grönlund et al. [27] which found *B. longum* to be the most common *Bifidobacterium* species present in infants. *B. longum* was also the most prevalent species in the antibiotic-treated infants. This result correlates with a recent finding by Mättö et al. [14], demonstrating that *B. longum* has a higher minimum inhibitory concentration (MIC) for ampicillin compared with other *Bifidobacterium* species. Thus, it appears that the antibiotic treatment itself may have selected for this species. One infant showed a decrease in numbers of species from three species at four weeks of age to one species at eight weeks of age. The species that showed the highest intensity in the DGGE gel after four weeks was replaced by another dominant species after eight weeks. These results are in agreement with a study by Favier et al. [26] in which the initially dominant bifidobacteria in two infants were replaced following the emergence of new species within the study time period. 

 Although less than half of the infants in this study were breast-fed, our study confirms previous reports that breast-feeding is positively associated with a more diverse bifidobacterial population compared with formula-fed infants [16, 28, 29]. *Bifidobacterium* species are considered among the most important beneficial bacteria in the human GIT [18, 19, 24]. A higher density of beneficial bacteria in infants may confer a degree of protection against childhood diseases. For example, bifidobacterial colonisation has been associated with a lower risk of atopy [21, 30, 31]. In a prospective study, children with lower numbers of fecal bifidobacteria and increased numbers of clostridia from the first weeks of life were more likely to become sensitized to common allergens [23]. In addition, it has been reported that differences in the gut microbiota during infancy may precede the onset of obesity. In this study by Kalliomäki et al. [32], the numbers of *Bifidobacterium* spp. were higher during infancy in children who remained at normal weight than those in children who became overweight. Factors such as breast-feeding, vaginal delivery, and the absence of antibiotic exposure would promote microbial diversity with higher numbers of bifidobacteria. 

In conclusion, the present study demonstrates that short-term parenteral antibiotic treatment of neonates causes a disturbance in the expected colonization pattern of bifidobacteria in the first months of life. Whether a delay in bifidobacterial colonisation could adversely influence health status, either in infancy or later life remains unknown. However, adequately powered prospective studies that document changes in the microbiota and incorporate long-term health status outcomes are needed to further our understanding of the sequelae, if any, of early neonatal antibiotic administration. Until such data are available, supplementation with bifidobacteria in this patient group cannot be justified.

## Figures and Tables

**Table 1:** Description of infant samples.

Sample	Sex*	Feeding**	Antibiotic treatment (days)	Type of Antibiotics^†^	Mode of delivery
A	M	B	+ (9)	Amp. + Gent.	Caesarean section
B^‡^	M	F + B	+ (5)	Amp. + Gent.	Caesarean section
C^‡^	M	B	+ (2)	Amp. + Gent.	Caesarean section
D	M	F	+ (2)	Amp. + Gent.	Vaginal delivery
E	F	F	+ (5)	Amp. + Gent.	Caesarean section
F	F	B	+ (2)	Amp. + Gent.	Vaginal delivery
G	F	B	+ (2)	Amp. + Gent.	Vaginal delivery
H	M	F	+ (2)	Amp. + Gent.	Caesarean section
I	M	F	+ (2)	Amp. + Gent.	Vaginal delivery
J	M	F	—	—	Vaginal delivery
K	M	F	—	—	Vaginal delivery
L	F	F	—	—	Vaginal delivery
M	F	F	—	—	Vaginal delivery
N	M	B	—	—	Vaginal delivery
O	F	B	—	—	Vaginal delivery
P	M	B	—	—	Vaginal delivery
Q	M	F	—	—	Vaginal delivery
R	F	F	—	—	Vaginal delivery

*M, male; F, female.**B, breast-feeding; F, formula feeding.^†^Amp. = ampicillin, Gent. = gentamicin. ^‡^ twins.

## Abbreviations

DGGE: Denaturing gradient gel electrophoresis
GIT: Gastrointestinal tract
MIC: Minimum inhibitory concentration
NICU: Neonatal intensive care unit.
